# Halophytes.tn: an innovative database for Tunisian halophyte plant identification, distribution and characterization

**DOI:** 10.1093/database/baab082

**Published:** 2022-03-19

**Authors:** Henda Merchaoui, Riadh Ksouri, Chedly Abdelly, Mohsen Hanana

**Affiliations:** Faculty of Sciences of Bizerte, University of Carthage, Zarzouna 7021, Tunisia; Laboratory of Aromatic and Medicinal Plants, Center of Biotechnology of Borj-Cedria, BP 901, Hammam-Lif 2050, Tunisia; Laboratory of Aromatic and Medicinal Plants, Center of Biotechnology of Borj-Cedria, BP 901, Hammam-Lif 2050, Tunisia; Laboratory of Extremophile Plants, Center of Biotechnology of Borj-Cedria, BP 901, Hammam-Lif 2050, Tunisia; Laboratory of Extremophile Plants, Center of Biotechnology of Borj-Cedria, BP 901, Hammam-Lif 2050, Tunisia

## Abstract

Halophytes.tn (http://halophytes.rnrt.tn/) is a web-based database of Tunisian halophyte species. Halophytes are salt-tolerant plants able to grow above 85 mM of salt, even up to 2 M as for Tecticornia spp. Tunisia, a North African country located on the Mediterranean border, covering ∼165 000 km^2^, harbors several types of saline habitats and biotopes where halophytes preferably vegetate. With ∼6000 worldwide and over 420 Tunisian species, halophytes represent a huge potential in several fields, including desalination, phytoremediation, agrofarming, medicinal use, industrial applications, pharmacology and even nanotechnology. We describe the practical and technical steps followed and bioinformatics tools used to conceive and design the first Tunisian halophytes database, enabling species identification and characterization. As a first version, information about botany, morphology, ecophysiology and biochemistry were provided for the identified species with their sites of growing in Tunisia, first step of biodiversity conservation, management and valorization. The database will be regularly maintained, updated and enriched to achieve the goal of whole Tunisian halophyte species and fit the needs of scientists and all category of users.**Database URL:** http://halophytes.rnrt.tn/

## Introduction

Halophytes could be defined in several ways, depending on whether considering the physiological, morphological, biological or ecological aspects (1–8). Broadly speaking, they are plants that are able to grow or preferably live in saline conditions. Ungar (8) defines them as plants that complete their life cycle in saline habitats and whose salt concentration is >85  mM NaCl according to Aronson *et al.* (1). They are further categorized into obligatory and facultative halophytes according to their salt need (9), hydro-halophytes (aquatic or wet conditions) and xero-halophytes (dry conditions) (10). Even though some halophytes obligatory need salt to grow, they reach ∼6000 species in the world, thriving in various types of climates and biotopes. Phylogenetically, halophytes are widespread among several families of angiosperms (1). Being distributed among so many different families indicates that this polyphyletic feature of halophytism suggests that salt-tolerance property could be transferred to glycophyte plants that are salt-sensitive species (11–15). Therefore, they constitute a valuable source of genes and molecular markers of salinity tolerance (16). Although halophytes have been considered initially as plant models for physiological and biological comprehension of salt adaptation mechanisms, more recently, they gained increased attention given their new agricultural applications (17–20), ecological (5, 21, 22) and phytoremediation potentialities (23), phytodesalinizing capacities (24, 25), nutraceutical, cosmetic and medicinal uses (26–28), bioenergy crops purposes (29–31), and even ornamental exploitation in urban cities (32, 33). The potentialities of their valorization and their emerging uses, in addition to their genetic and physiological properties, are currently the subject of considerable research. In Tunisia, at the Center of Biotechnology of Borj-Cedria, particularly the Laboratory of Extremophile Plants, we have been working on halophyte species for 30 years. More than 500 scientific papers, chapters and books have been published, mainly headed by Professor Abdelly Chedly. Although halophytes have been deeply studied under several aspects (i.e. physiology, ecology, biochemistry, biology, molecular, etc.), the key identification steps of these species are still poorly understood and often confusing. Indeed, missing a comprehensive and single definition of halophytes, students and young scientists do frequent misidentifications and find difficulties in recognizing plant samples within Tunisian sites. Therefore, due to the lack of precise information and updated data, the conception of halophytes database became of urgent need. The history of halophyte plants databases started in 1974 with Mudie who published a list of 550 species (34), followed by Aronson *et al.* in 1989 (1) who made a catalog of 1560 species belonging to halophyte category. Afterward, Menzel and Lieth compiled a list of 2600 species on CD electronic support format (35). Later on, a series of country-related databases have emerged in format of papers, book chapters or book compilations such as ‘Halophytes in China’ (36–38), ‘Sabkha ecosystems’ from Arabian peninsula and adjacent countries, as well as from West and Central Asia, Pacific, Africa and Southern Europe, East Mediterranean and the Americas (several authors in ‘Tasks for vegetation science’ book series (39–47)), ‘Halophytes in the state of Qatar’ (48), ‘The Halophytic Flora of Syria’ (49), ‘Romanian Salt Tolerant Plants’ (50), ‘Database of halophytic vegetation in Serbia’ (51) and ‘Database of halophytic and littoral vegetation of Ukraine’ (52). Thereafter, much more sophisticated and web hosted databases appeared, such as eHALOPH (53) (http://www.sussex.ac.uk/affiliates/halophytes/) and extremeplants (http://extremeplants.org/), which is more dedicated to extremophile plants but also includes halophytes class. In Table 1, the known and accessible lists and databases of halophyte species in the world are mentioned. As far as Tunisia is concerned, halophytic species’ populations in their natural habitats are to be studied, and tremendous efforts need to be embedded in this field. Therefore, we started with recording halophyte species features, saving and compiling them in order to gradually design a Tunisian halophytes list, ending to a comprehensive database.

**Table 1.** Available halophyte databases and lists in the world

Database/list name	Reference	Plant country/region
List by family, genus and species	(34)	USA
HALOPH: a database of salt-tolerant plants of the world	(1)	Worldwide
List by family, genus and species	(9)	Mediterranean
List by family and genus	(36–38)	China
List by family, genus and species	(57)	Worldwide
List by family, genus and species	(58)	Palestine
List by family, genus and species	(45)	Pakistan
List by genus and species	(59)	Qatar
List by family, genus and species	(49)	Syria
List by family, genus and species		Romania
eHalophytes	(53)	Worldwide
List by family, genus and species	(60)	Jordan
HALOPHYTE Database Vers. 2.0	(35) on CD	Worldwide

Tunisia is a North African country with an area of 163 610 km^2^; and has diverse types of climate; ranging from Mediterranean on the northern coast (humid and sub-humid) to Saharian towards the south (semi-arid to arid desert). Annual rainfall ranges from 500 mm in the north to <100 mm in the desert south. Some information is available about saline biotopes in Tunisia but rarely offers details about plant communities and species. Salt-affected soils in Tunisia cover ∼1.5 million hectares, representing 10% of the country’s surface area (54). In Tunisia, halomorphic soils are found within diverse types of biotopes, namely, salt lakes known as ‘sabkhas’ and ‘chotts’, saline inland basins, coastal sandy lands and marshes where halophytes could be found and grow. Unfortunately, climate change, anthropomorphic pressure, soil erosion and overexploitation through industrial and urban activities led to natural resource loss and biotope degradation. Our database aims therefore to highlight the identification, characterization and valorization of halophyte species mainly found in Tunisia, thus contributing to the diversity conservation and halophytic habitat preservation and opening windows for halophyte sustainable exploitation and new potentialities. Therefore, it will highlight the role of halophytes in desalination process, food security and therapeutic uses. Also, it will serve as a reference information for both students and researchers, which will help them in their methodological approaches, taxonomic difficulties, teaching and coursing requirements and also ultimately gives basic knowledge for any kind of reader or user.

## Materials and methods

### Survey, sampling and botanical identification

**Figure 1.**
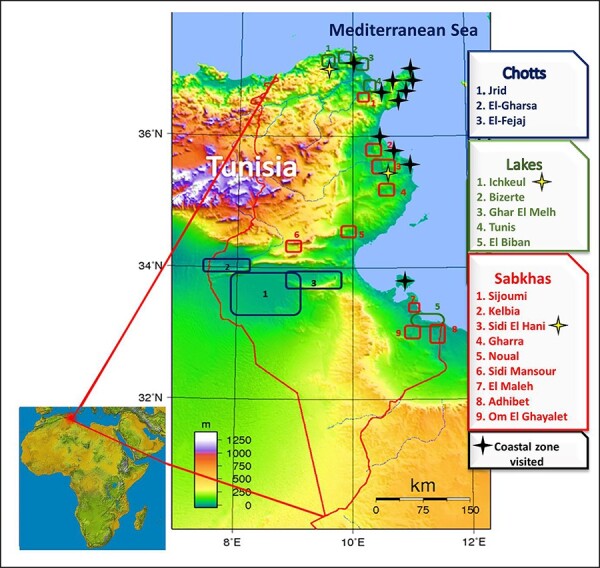
Strategic geographical location of Tunisia in the Mediterranean basin and map of the main potential halophyte locations in Tunisia (Chotts, lakes, Sabkhas and coastal zones).

We firstly identified potential and suitable sites where halophytes should logically be found and grow. Thirteen sites within coastal zones, ‘chotts’, lakes and “sabkhas’, were selected for halophyte species sampling (Figure 1). Whereupon, prospections were planed according to seasons and geographical positions of the habitats, for which pedo-climatic characteristics and Global Positioning System (GPS) coordinates were determined and saved. All locations selected in this study did not require specific permission. For each visited site, in order to obtain accurate information on community composition and to obtain a clear view about area and individuals, we started sampling with photographs, avoiding fastidious work of systematic and transect methods. Thus, we first took photographs to obtain a global overview of the plant community, followed by close-up views of each single species, focusing on all organs, particular structures (i.e. salt glands, etc.) and developmental stage (flowering, particular characteristics over season, etc.). For some sites, videos were taken by a camera to provide a better sense of features and conditions of the biotope. Whenever possible, aerial parts (stems, leaves and seeds) of plant were collected in plastic bags for later genomic DNA and messenger RNA extractions, germination experiments and seed databank obtention; however, as a first version of the database, no voucher specimen has been conserved nor planned yet. Samples of soil were taken for analysis in order to confirm their sodic characteristics. Then, all field data and notes were organized and saved as files for further analyses. Details about what format to use depend on the type of data (i.e. jpeg for image, word for text and excel for spreadsheet). This data transcription is accompanied by detailed documentation and bibliography. Finally, each photo posted on the website database is accompanied by the title, the identification of the species, the GPS location of the site, the date taken and the name of the photographer. Basically, our survey does not include mapping vegetation populations nor quantifying the plant community but aims to identify and characterize halophytic species within their natural habitats, while providing global and outstanding features of vegetation population and site. Initially, we carried out bibliographical research and ethnobotanical inventory of Tunisian halophytes whose number could potentially reach 420 species (50, 55). This first step made us more familiar with halophyte species recognition and identification. The main botanical guides and references that have been used are listed in Table 2. Usually and whenever possible, for known species, identification is made *in situ* upon collection; however, for unknown species and confirmation of taxonomy, specimen identification is undertaken at a later date using halophyte references, taxonomic keys (published in books, journals and CDs) and flora identification tools (online sites and databases) and by comparison with herbarium specimens and consultation with botanical experts. Misidentification should be avoided by checking several references, databases and with experts. Species listed as ‘threat’ according to the red list of the International Union for Conservation of Nature (https://www.iucnredlist.org/) are not collected but just identified and recorded in their site.

**Table 2.** Main sites, books and references consulted for halophytes listing and identification

	Name/title	Reference/web address
Databases	Flore du Maghreb	https://www.ville-ge.ch/cjb/flore/html/QSv2-ALL.htm
	IdentiPlante	https://www.tela-botanica.org/
	Pl@ntNet	https://identify.plantnet.org/fr
	The Plant List	http://www.theplantlist.org/
	eHALOPH	https://www.sussex.ac.uk/affiliates/halophytes/
	Plantes & Botanique	https://www.plantes-botanique.org/
	Société botanique de France	https://societebotaniquedefrance.fr/
Botanical books	La Flore de la Tunisie	(61)
	Catalogue synonymique de la Flore de Tunisie	(62)
	Index synonymique—Flore d’Afrique du nord	(63–65)
Ethnobotanical books	Les plantes dans la médecine traditionnelle tunisienne. Médecine traditionnelle et pharmacopée.	(66)
	La flore succincte et illustrée des zones arides et saharienne en Tunisie	(67)

### Dataset and processing

**Figure 2.**
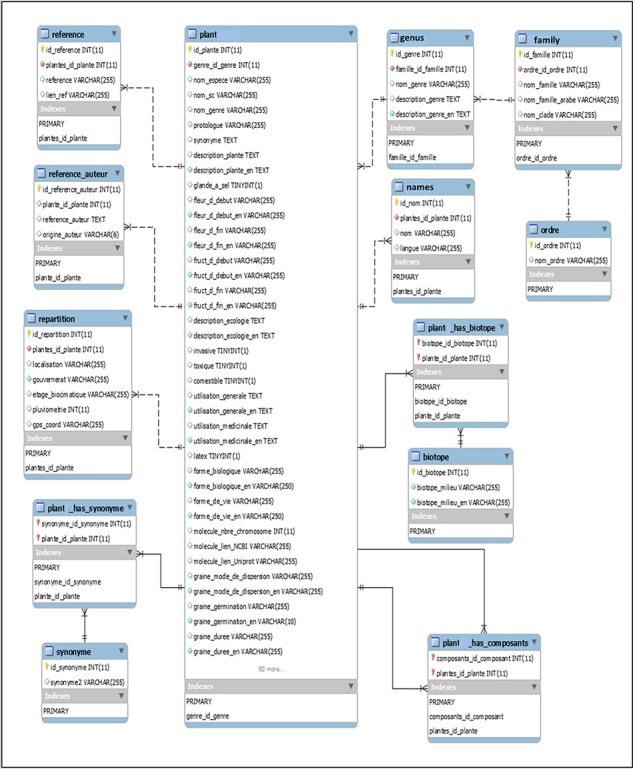
Database structure: main tables and their relationships (mysql-workbench-community-6.3.10-winx64).

**Figure 3.**
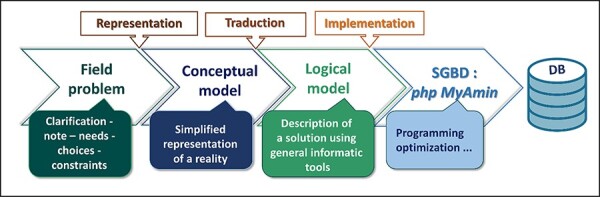
The four global steps that led to the Halophytes.rnrt.tn database conception.

The database was initially conceived with the aim to collect and share publicly all knowledge and available information on the Tunisian halophyte species held by survey and literature consultation. The dataset of Halophytes.rnrt.tn database is organized around and onto the species for which several descriptors and determinants are given and have been made available for the user. These parameters are first treated with Excel software and tools previous to their transfer into CSV comma separated value extension file. Thus, each species has a wide range of features and properties such as region, biotope, biology and ecology data, and botanic and morphological data illustrated by photos showing general view and particular organs and tissues, common names, synonyms, associated phytochemicals, ethnobotanical and potential uses, and bibliographic references. Figure 2 shows the main tables and their relationships. However, some of these features such as molecular data are not yet accomplished. Schematically, the approach followed to design the database is based on the following four successive steps: strategic analysis and planning, conceptual modeling, logical modeling and implementation (Figure 3). Each of these processing phases has its own means and appropriate tools that will ensure their achievement. Besides, in the Mediterranean basin, the overall halophytic flora is ∼600–800 species, which represents 3–4% of the overall phanerogamic flora (9). In Tunisia, 420 halophyte species have been documented, belonging to 230 genus, 69 families and 28 orders (56). As a first step, we recorded information only for 30 species belonging to 14 families; furthermore, researches are underway to widen the content of our database and increase the number of species.

## Results and discussion

### Database design, implementation and web site interface

**Figure 4.**
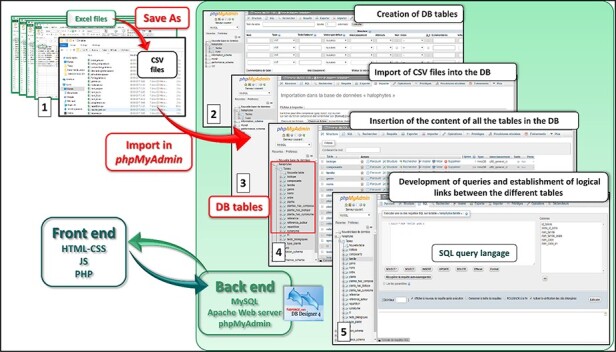
Web development strategy of Halophytes.rnrt.tn database.

Data collected from different sources were saved as separate files using appropriate filters. Initially, all the data were manually stored in Microsoft Excel tables, which are then converted to CSV files. Then, they are migrated to MySQL database (version 2.5.21) using phpMyAdmin administration tool. In fact, the back-end architecture is based on Apache web server processing requests (version 2.4.33), serving web content via HTTP. This Apache web server communicates with the MySQL relational database to get all the needed information about the plants. The database model was designed and created using DBDesigner 4. The front-end side is however developed using PHP (version 5.6.35), JavaScript, CSS and HTML (Figure 4). Halophytes.tn was designed to provide the best user experience using Google Chrome, Mozilla Firefox and Opera web browsers. During the development stage, the website was hosted locally to accelerate the development phase. After this stage, the hosting of the website was carried out by our institution (Center of Biotechnology of Borj-Cedria), which is now responsible for the website maintenance. Halophytes.rnrt.tn website allows for anyone interested in Tunisian Halophytes plants, to easily recover basic and specialized information about them.

### Database structure

The database has 13 tables on data storage and selection.

Recorded data are primarily based on key species determination through taxonomic characters and information which refer to order, genus, family, names and other features that are organized in tables and schematized with their relationships (Figure 2). The ‘plant’ table describes the botanical, ecological, biological, physiological and biochemical characteristics of the species, with a description of seed morphology and physiology. This table, which is linked to all other tables, contains also data about ethnobotanical and medicinal uses of plants in Tunisia and all over the world, their biological effects and the body systems and organs on which each species acts. It also shows the results of some laboratory analyses, especially the antioxidant activities. The table named ‘repartition’ contains data of each site and its geographical coordinates in decimal degrees, which depicts the visualization of the data on a geographical map. For the taxonomy of each species, other tables were designed to include order, family and genus, which were also interconnected and linked to the principle table ‘plant’. The purpose of the table ‘biotope’ is to provide the list of species in a determined site (coastline, ‘sabkhas’, desert, ‘chotts’). The table named ‘plant-has components’ includes molecular content information about each species, i.e. polyphenols, fatty acids, amino acids, vitamins, etc. Finally, two other tables for references containing information on the bibliographic references (both Tunisian and foreign ones) related to each species are added. The configuration of the database structure was built as much as possible with related data in forms of connected tables and entities.

### User interface

#### Database logo identity

**Figure 5.**
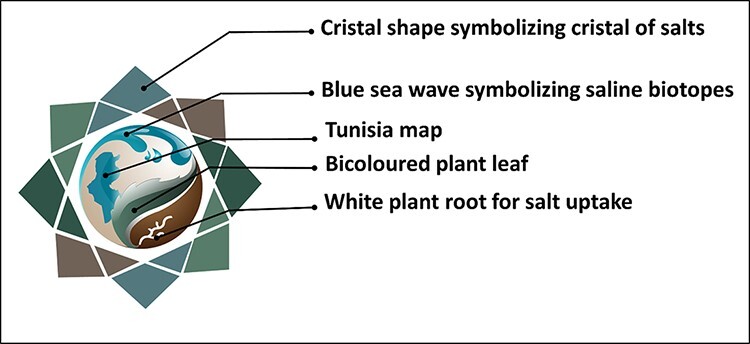
Logotype meaning of the Halophytes.rnrt.tn database.

In order to build a brand recognition for our website, a logotype was first designed using Adobe Illustrator (version CS6) and Adobe Photoshop software (version CS3). The logo was designed based on assembly and combination of a set of symbols, forms and colors with scientific meaning (Figure 5). The main shape of the logo is a crystal, in reference to the salt crystal. Its center is schematized by a circle containing a blue sea wave representing the saline biotope, in addition to the map of Tunisia, a bicolored leaf symbolizing halophytic plants and a white root designating the uptake of salts. All colors used are directly related to halophyte colors, mainly the glaucous green. White color is used to represent halophyte environment, brown color for the soil and the blue color for the seawater and all kinds of saline biotopes.

#### Web user interface

The web interface comprises a collection of web pages related to the data (Figure 6). The home page is available in three languages—Arabic, French and English (Figure 6A), in order to let visitors choose their preferred language, whether they are researchers, graduate students or even unfamiliar with plant biology users. The web pages are actually in two languages—English and French. It contains the main objectives of this database and other links to access each species and contacts (Figure 6B).

**Figure 6.**
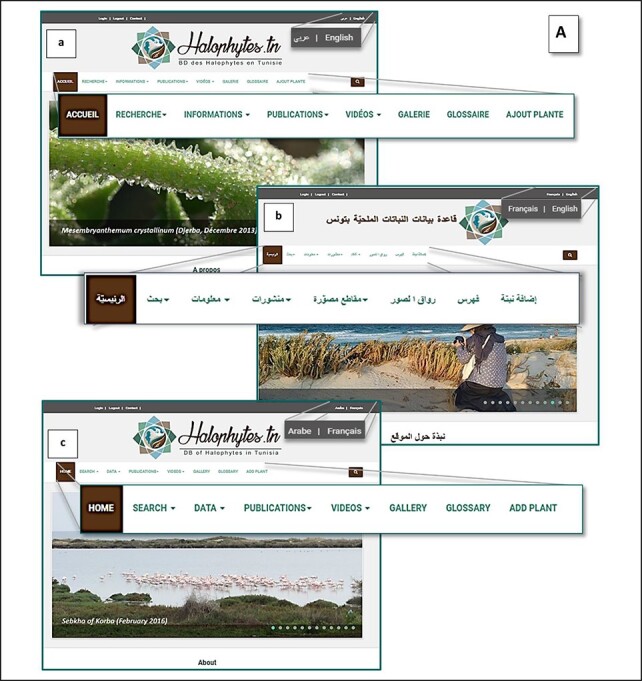
(A) Home page of the Halophytes.rnrt.tn website available in three languages: a: French, b: Arabic, c: English.

**Figure 6.**
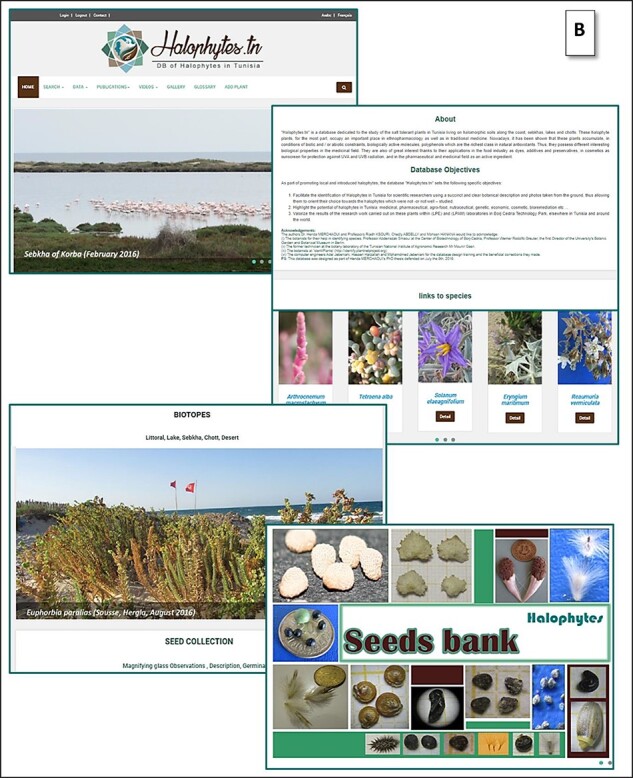
(B) Other topics and links of the home page.

#### Search criteria

**Figure 7.**
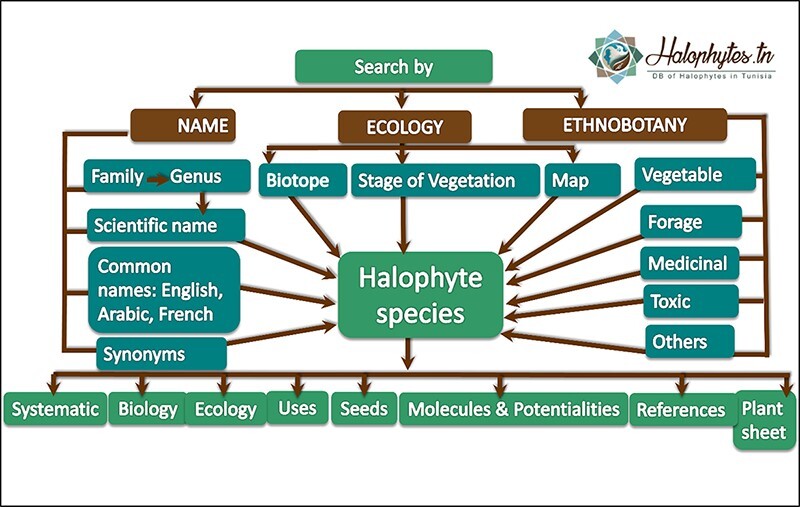
Framework of the Halophytes.rnrt.tn database showing the different search criteria in the homepage.

**Figure 8.**
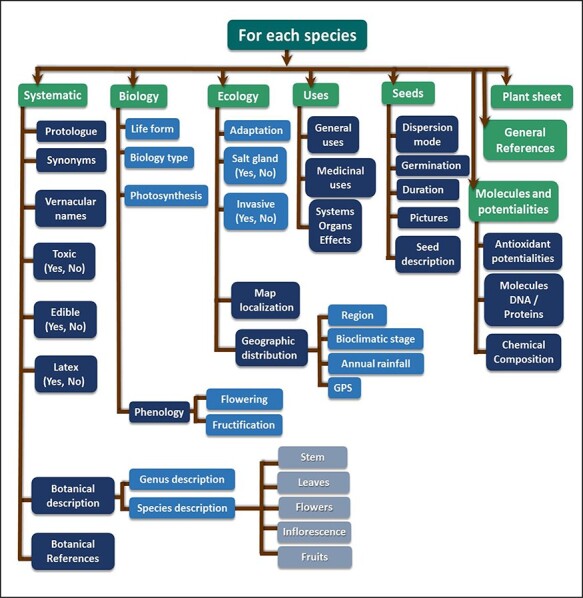
Framework of several search criteria for each species.

**Figure 9.**
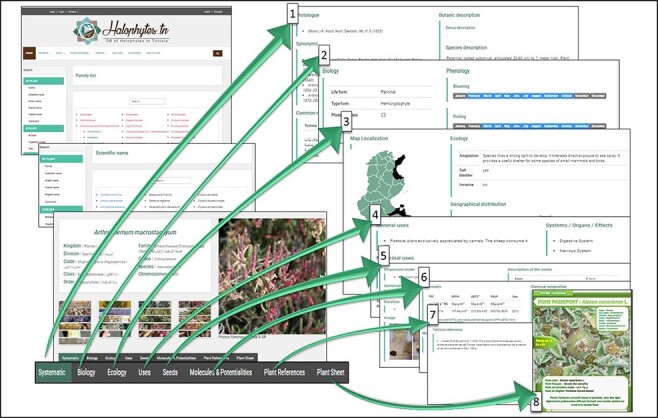
Organization of the different webpages related to each species: 1: systematic information and botanic description; 2: biological data and phenology; 3: ecological data and geographical distribution; 4: general and medicinal uses; 5: seeds data; 6: molecules and potentialities; 7: references; 8: plant sheet.

Halophytes.rnrt.tn database was established to enable users to browse, search, view and identify easily and quickly species, with clear, succinct and effective data and pictures (Figures 7 and 8). Halophytes.rnrt.tn database could be navigated by five sections: search (by family, scientific name, Arabic name, French name, English name and synonyms), data related to saline environments (phylogeny, physiology and phytochemistry), gallery, videos (visited sites) and glossary (botanic terms). Each species have eight webpages (Figure 9): the first one contains systematic information and botanic description (genus and species description) accompanied by their references. The second page shows biological data (life and type form, photosynthesis pathway) and phenology (blooming and fruiting). The third page shows ecological data (localization mapping, ecological adaptation, presence or not of salt glands, invasive or not) and geographical distribution (region, annual rainfall, bioclimatic stage and GPS coordinates). The fourth page presents general and medicinal uses, effects and the body systems and organs on which plant acts. The remaining pages contain information related to seeds, bioactive molecules, references and a plant sheet summarizing all the information relating to each species. The ‘Add plant’ section gives the possibility to users to add a new plant (**Supplemental file 1**). Each new contribution to the database will be rewarded with a certificate delivered by the database managers (**Supplemental file 2**).

#### Website maintenance and database update

The website will be regularly maintained and secured through checking for issues and mistakes. Therefore, to ensure the website is correctly working, serial tasks needs to be performed like check-ups on all devices and browsers to see if it displays correctly, normal pages loading assessment, spams removing, properly running of software and plugins, fixing broken links, user testing. In addition, both automatic and manual backups should be performed to restore the website information if needed. Halophytes.tn is regularly maintained and updated to meet the needs of scientists and all categories of users. For a new species contribution, whether done by the administrator or a foreign registered user, minimum information (taxonomy, botany, morphology, localization, etc.) should be supplied, otherwise, new data about already existing species are welcome. Besides, any update should be first authenticated and then approved by the administrator and acknowledged by a certificate of database contribution. We aim to continuously update Halophytes.tn, adding new and relevant information (omics, propagation techniques, medicinal and agro-industrial applications, etc.) or services (seed supply, vitroplants and plant cuttings delivery) on Tunisian halophytes, expanding and improving the database for maintaining a high-quality, up-to-date database.

## Conclusion and perspectives

We have generated the first web-based database that provides researchers and users with resources and information related to Tunisian salt-tolerant plants. Halophytes.tn website allows user to easily recover basic and specialized information about Tunisian halophytes, to browse, search, view and identify species with all clear, succinct and effective data with plant pictures and several features. Tunisian halophytes are a valuable source of salt-tolerant plants that could be exploited in a multitude of applications, mainly desalination and phytoremediation processes, agro-industry, medicine and pharmacology. In perspective, the number of halophyte species in Halophytes.rnrt.tn database is expected to increase and reach the Tunisian Halophytes pool. Moreover, incorporation of omics data and innovating bioinformatics tools, with the addition of new devices such as biological material collections (i.e. seeds, plant cuttings and vitroplants), cDNA libraries and gene bank would enhance its attractability. Collaborations with other laboratories, scientific associations and academicians would be planned to enlarge the database to Mediterranean and worldwide levels.

## Supplementary Material

baab082_Supp
