# Supplementary material for: Halophytes.tn: an innovative database for Tunisian halophyte plant identification, distribution and characterization
Source: Database (Oxford). 2022 Mar 19;2022:baab082. doi: 10.1093/database/baab082 (PMC9216540; doi:10.1093/database/baab082)
Supplement: baab082_Supp [file baab082_supp.zip › supplementary files.docx]

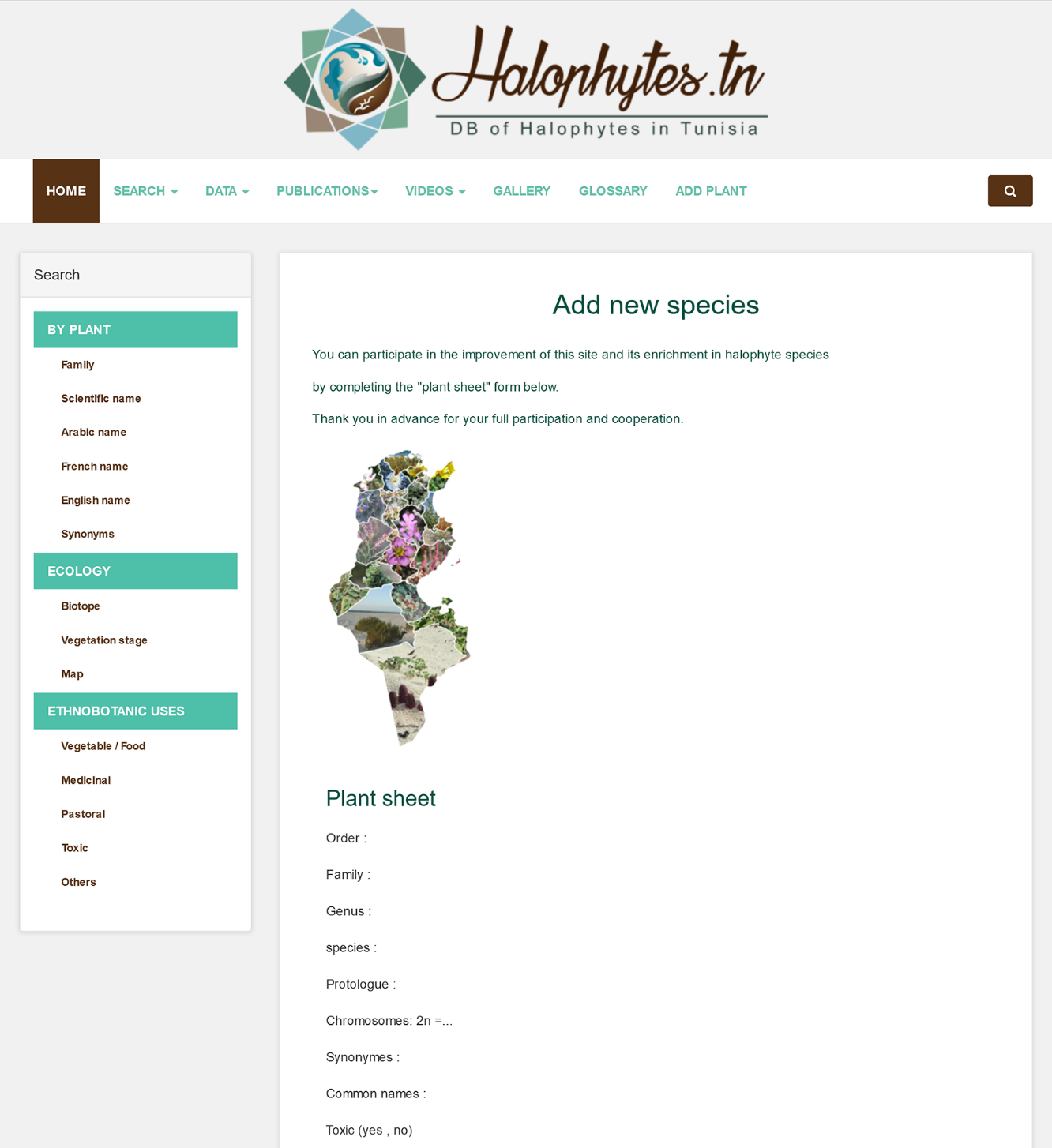
**Fig. 10.** Add plant page.


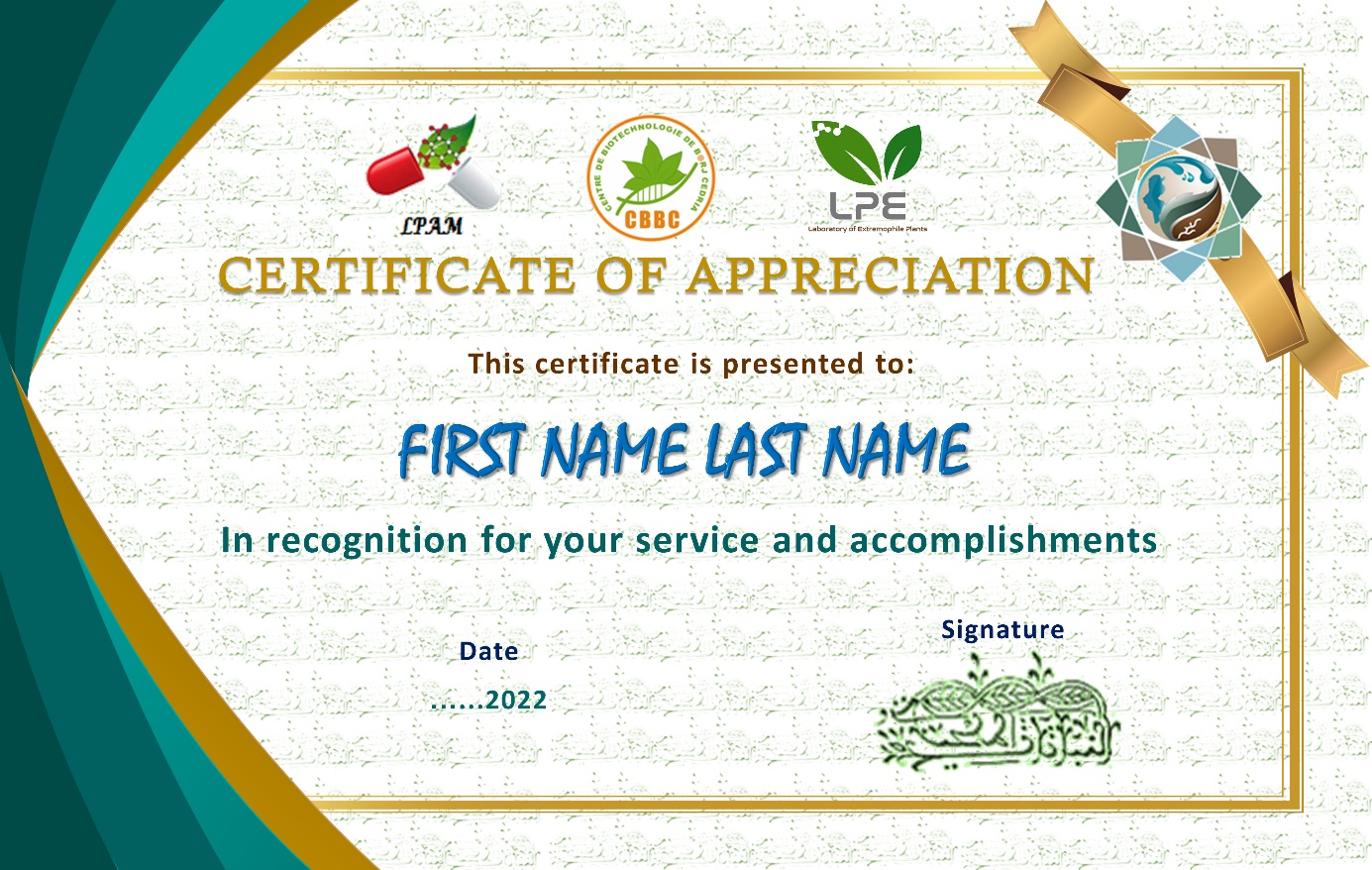
**Fig. 11.** Certificate of contribution.

**Table 3**

The fourteen families of the thirty-recorded species in the Halophyte.tn database.

| **Family** | **No. of species** | **No. of records** |
| --- | --- | --- |
| **Aizoaceae** | 5 | 3 |
| **Amaranthaceae** | 50 | 7 |
| **Apiaceae** | 11 | 2 |
| **Asteraceae** | 37 | 5 |
| **Brassicacea** | 15 | 1 |
| **Cynomoriaceae** | 1 | 1 |
| - **Euphorbiaceae** | 6 | 1 |
| **Fabaceae** | 38 | 2 |
| - **Nitrariaceae** | 1 | 1 |
| - **Plumbaginaceae** | 8 | 1 |
| **Poaceae** | 58 | 1 |
| - **Solanaceae** | 9 | 1 |
| - **Tamaricaceae** | 7 | 2 |
| - **Zygophyllaceae** | 5 | 2 |
| **Total** | **251** | **30** |
